# Proper Management of the Proximate Cutting and Grinding Surfaces of the Teeth

**Published:** 1880-06

**Authors:** George B. McDonald


### ARTICLE IV.

### *Proper Management of Proximate, Cutting and Grinding Surfaces of the Teeth.*

BY DR. GEO. B. MCDONALD.

In consideration of failures in our attempts to stay the ravages of caries in the proximate surfaces of the human teeth, I have decided to devote this paper to the showing of the causes of failures, with a view of refilling cavities on such principles as to guard the fillings from failure in the future. As the cutting and grinding surfaces of the teeth are often involved with the proximate ones, I deem it proper to include them in the few remarks I shall make upon this subject.

It will be sufficient for my present purpose to describe the proper preparation and filling of the proximate surfaces of a right-upper central incisor and right-superior first bicuspid, where the cutting surface of one and the masticating surface of the other are involved, they having been previously filled and now requiring to be refilled. Before giving a description of such an operation as should be performed upon the central incisor referred to, let us first ascertain the cause or causes of the failure of the fillings previously introduced. If we succeed in finding this we shall undoubtedly be able to see our way clear to successfully fill this tooth, so that the operation cannot fail again. After a careful examination of the tooth in question we arrive at the following conclusions: *First*, We did not have room enough, laterally, to fill the tooth properly, as we wedged the same day we filled it, when we should have pressed it and the adjoining tooth apart for one week or more, as circumstances might indicate. *Second*, We did not properly anchor our filling in the cervical, palatal and labial walls of the tooth. *Third*, We did not bevel the margins of the cavity thoroughly, especially at the cervical margins, leaving a frail and partially disintegrated structure, which continued to disintegrate after the fillings were inserted. *Fourth*, We packed our gold at the cutting edge in enamel, which shows that there were checks in the cutting edge of the tooth, and these checks caused the cutting edge to split off when exposed to the forces used in biting hard substances. *Fifth*, We discover that too large pieces of gold were used, and not perfectly impacted. Only one hour was spent on them with an automatic or hand mallet, when, to make a perfect operation, we should have spent a much longer time, even with the use of the electro-magnetic mallet, in impacting small pieces of gold foil into the cavity. *Sixth*, The gold was not finished down perfectly even, especially at the cervical margin, thereby allowing lodgments of destructive agents along the margin of the filling which caused disintegration of the enamel. These are six good and satisfactory reasons why our fillings failed, any one of which was sufficient.

With the knowledge gained in ascertaining why we failed, we will proceed to refill this tooth, with the intention of preserving it for twenty years or more. Having wedged it apart from the adjoining teeth from one-sixteenth to one-eighth of an inch, and having kept it in this position one week or longer, or until all soreness that has been induced in the peridental membrane by the wedging shall have subsided, we now apply the rubber dam; and, taking a proper shaped corundum wheel, cut away that portion of the cutting edge of the tooth yet remaining between the cavity in the mesial and the one in the distal proximate surface more or less, as the case may require. Now, with proper shaped stone-cut engine burrs, we make retaining grooves through the cutting edge of the tooth, extending them to cervical, palatine and labial walls. With bud-shaped enamel burrs we bevel or properly shape the entire orifice of the now compound cavity, removing every particle of disintegrated tissue, and with a strip of No. O emery cloth polish off the external margins. With the hot-air syringe we blow out the chips that may be in the cavity and pronounce it ready for the gold. Now take one sheet of cohesive gold foil (No. 4,) fold it four times with a nickel-plated spatula, and with a pair of foil shears cut into strips one-sixteenth and one-eighth inches wide; take a strip of the narrow gold, anneal it slightly, and with a properly-pointed hand instrument anchor it instarting points made along the cervical wall of the cavity. Now with the electro-magnetic mallet we proceed slowly, surely and perfectly to pack these small pieces of gold foil against the walls until we have restored the tooth to its original contour with solid gold, perfectly uniform in density from center to circumference, which will stand the force of incising food like cast steel, and cannot possibly be dislodged from its position without the breaking of the tooth at or near the neck. We now finish this filling with properly shaped separating saws, files and strips of emery cloth till it is ready to polish. The polishing is effected by the use of strips of crocus cloth and whiting on linen tape, with the assistance of the dental engine, flexible rubber cones and wheels.

On an examination of the right-upper bicuspid, which has previously been filled on its mesial and distal proximate surfaces, we find the filling partially out and the structure extensively decayed around the fillings, which would fall out were it not for the adjoining teeth. We find all the causes of failure that were present in the case of the incisor with two additional ones, namely: We left an organic fissure on the grinding surface of the tooth, which should have been opened into with a small, thin corundum wheel and the two proximate cavities connected. We also find, upon an examination of the gold, that we had used too large pieces in filling, as cylinders, pellets and ropes of gold foil twisted up with our fingers. On taking the filling apart the gold separated easily, and each filling is made up of different large pieces of gold pressed loosely against each other, there being no cohesion of the gold—the condition that must exist in order to get the best results.

After wedging this tooth the same as the incisor, we apply the rubber dam and proceed to prepare for filling. When nearly ready we have a compound cavity presented, extending from the cervical wall of the mesial surface through the fissure to the cervical margin of the distal surface. We find that the walls of the tooth left standing are partially separated from each other, and that they are so attenuated that if left standing they will be likely to break off. We are more certain that this might occur when we notice that each cusp is quite long, its antagonist having long cusps also. Our past experience has taught us, with this condition of things one or both of the cusps may be split off by the occlusion of the inferior teeth; so we cut off the cusps, using corundum wheels of suitable form. After removing all the decayed portion we make starting points in the cervical walls and anchoring grooves along the palatine and buccal walls with a small stone-cut burr, having previously finished the entire margin of the cavity with a fine, sharp, bud-shaped burr, being sure not to leave a particle of disintegrated enamel at the orifice, where the gold and tooth tissue will be in contact and exposed to destructive agents. We proceed to fill, as before, by packing narrow, thin strips of gold into the starting points with a proper-shaped hand plugger, completing the operation with the electro-magnetic mallet, being sure to condense each piece thoroughly against the enamel and throughout the entire filling until we have restored the contour of the tooth, thereby making a gold crown with such gold cusps as can no more be split by the occlusion of the inferior teeth than could a crown of steel. Having finished this operation as we did the incisor, it is absolutely necessary that the adjoining teeth should knuckle up to the gold one, so as to come into perfect contact, to prevent food from getting between the teeth, thereby irritating the gums, as is so often the case where V-shaped separations are made between them. If we have spent several hours on these operations, and have charged thirty or fifty dollars each for them, it will be infinitely better and cheaper for the patient than any other method.

I will here give a simple illustration of the principles involved in the method of operating which I have just given. If a barrel were hooped with common twine it would be a very weak vessel, and the staves would separate when but little force was brought against them. When a barrel is hooped with iron the staves become a unit, as it were. Such a barrel can withstand tons of mechanical force without being broken. The ordinary method of filling frail and extensively decayed teeth with cylinders, pellets and ropes of gold foil twisted up with the fingers and packed with automatic or hand mallet is to be compared to the twine-hooped barrel, while the process I have heretofore described is to be compared to the barrel with the iron hoops. A tooth restored according to the method I describe will stand any necessary force without being split apart, because the enamel is supported by a solid band or hoop of gold as strong as steel. There are those who argue against cutting away too much tooth-structure. I assert, without fear of successful contradiction, that there are ten failures for the want of cutting away enough frail tooth-structure, to be replaced with rolled gold, where there is one from too much cutting.

"Haste makes waste," is an old maxim, and in nothing can it be better illustrated than when performing operations on the natural teeth. Hasty work is usually very imperfect, while operations upon which sufficient time is spent are more likely to be successful. I will here say that the value of clinics, to illustrate at our meetings that which relates to operative dentistry, cannot be over-estimated. Finally, I would say that if we practice in accordance with the principles herein stated our efforts will usually be crowned with success, and the teeth of our patients saved and restored to their former usefulness for many years.—*Trans. American Dental Association.*

